# Computed Tomography in Adults with Bronchiectasis and Nontuberculous Mycobacterial Pulmonary Disease: Typical Imaging Findings

**DOI:** 10.3390/jcm10122736

**Published:** 2021-06-21

**Authors:** Sabine Dettmer, Felix C. Ringshausen, Jan Fuge, Hannah Louise Maske, Tobias Welte, Frank Wacker, Jessica Rademacher

**Affiliations:** 1 Department of Diagnostic and Interventional Radiology, Hannover Medical School, Carl-Neuberg-Str. 1, 30625 Hannover, Germany; dettmer.sabine@mh-hannover.de (S.D.); Hannah.L.Maske@stud.mh-hannover.de (H.L.M.); wacker.frank@mh-hannover.de (F.W.); 2 Department of Respiratory Medicine, Hannover Medical School, Carl-Neuberg-Str. 1, 30625 Hannover, Germany; Ringshausen.felix@mh-hannover.de (F.C.R.); fuge.jan@mh-hannover.de (J.F.); Welte.Tobias@mh-hannover.de (T.W.)

**Keywords:** bronchiectasis, diagnostic imaging, nontuberculous mycobacteria, computed tomography

## Abstract

Among patients with bronchiectasis, nontuberculous mycobacterial pulmonary disease (NTM-PD) ranged between 1 and 6% and it is suspected that its prevalence is underestimated. Our aim was to evaluate differences in computed tomography (CT) features in patients with bronchiectasis, with and without NTM-PD, in order to facilitate earlier diagnosis in the future. In addition, we evaluated longitudinal changes after successful NTM-PD treatment. One hundred and twenty-eight CTs performed in adults with bronchiectasis were scored for the involvement, type, and lobar distribution of bronchiectasis, bronchial dilatation, and bronchial wall thickening according to Reiff. In addition, associated findings, such as mucus plugging, tree-in-bud, consolidations, ground-glass opacities, interlobular thickening, intralobular lines, cavities, and atelectasis, were registered. Patients with NTM-PD (*n* = 36), as defined by ATS/IDSA diagnostic criteria, were compared to bronchiectasis patients without NTM-PD (*n* = 92). In twelve patients with an available consecutive CT scan after microbiological cure of NTM-PD imaging findings were also scored according to Kim and compared in the course. In patients with NTM-PD, there was a higher prevalence of bronchiectasis in the middle lobes (*p* < 0.001), extended bronchiolitis (*p* = 0.032) and more small and large nodules (*p* < 0.001). Furthermore, cavities turned out to be larger (*p* = 0.038), and walls thickened (*p* = 0.019) and extended (*p* = 0.016). Patients without NTM more often showed peripheral ground-glass opacities (0.003) and interstitial changes (*p* = 0.001). CT findings decreased after successful NTM-PD treatment in the follow-up CT; however, without statistical significance for most features (*p* = 0.056), but bronchiolitis was the only significantly reduced score item (*p* = 0.043). CT patterns in patients with bronchiectasis and NTM-PD differ from those of patients with bronchiectasis without NTM-PD, although the findings are non-specific radiological features. Follow-up CT findings after microbiological cure differed interindividual regarding the decline in imaging features. Our findings may help practitioners to identify NTM-PD in patients with bronchiectasis. Further research is needed regarding the use of CT as a potential imaging biomarker for the evaluation of treatment response.

## 1. Introduction

Nontuberculous mycobacteria (NTM) are ubiquitous environmental bacteria that cause opportunistic infection in humans [1]. Pulmonary disease, due to NTM (NTM-PD), is a rare, chronic, and slowly progressive disease [1]. The most common species that causes this disease is mycobacterium avium complex (MAC), followed by mycobacterium abscessus complex. The incidence of NTM-PD is expected to increase worldwide. In the USA, incidence increased from 3.13 per 100,000 in 2008 to 4.73 per 100,000 in 2015, particularly among women and older age groups [2]. The use of a machine learning-based algorithm applied to German statutory health insurance claims data predicted a 9-fold increase in incidence rates, suggesting that a relevant number of patients with NTM-PD remains undetected [3].

Bronchiectasis is a significant disease and has a substantial impact on patients’ morbidity and mortality, as well as healthcare utilization [4,5]. Chronic airway infection plays a key role in the pathogenesis of the disease, sustaining a vicious cycle of inflammation and structural damage [6,7]. Among patients with bronchiectasis, NTM-PD ranged between 1 and 6% with higher rates in the US bronchiectasis registry [8,9,10], and it is assumed that the prevalence is severely underestimated [11]. In particular, patients with bronchiectasis have been found to have a substantially increased risk of NTM-PD; however, there is still a need to improve awareness among physicians treating patients with bronchiectasis [12].

European guidelines for the management of bronchiectasis do not recommend regular testing for NTM. Testing, as part of the initial work-up of bronchiectasis, is in fact only recommended in case of suggestive clinical features [13]. The question is, how do you identify patients with previously unrecognized NTM-PD? A survey among physicians managing patients with bronchiectasis showed that NTM testing needs to be improved, in particular with regard to NTM screening prior to the initiation of macrolide maintenance therapy [12].

Two forms of NTM-PD are generally recognized: the slowly progressing nodular-bronchiectatic form, most commonly in post-menopausal women, and the fibrocavitary form, which progresses more rapidly and occurs predominantly in middle-aged (former) smokers with a history of underlying lung disease [14]. Characteristic computed tomography (CT) findings of NTM-PD are bilateral bronchiectasis, multifocal bronchiolitis, lobular consolidation, and cavitation [15]. Radiologic presentation is similar to post-primary tuberculosis; however, NTM infection usually progresses more slowly than active tuberculosis [16]. Chest CT has been used in a wide variety of clinical settings: diagnosing, monitoring, and determining the timing of treatment initiation, and evaluation of treatment response in NTM-PD [15,17]. Today, chest CT is considered the gold standard for establishing the diagnosis of bronchiectasis and is obligatory for inclusion in national and international bronchiectasis registries [6,7]. The use of HRCT findings could help differentiate between bronchiectasis patients with and without NTM-PD.

The purpose of this study is to further evaluate specific findings of NTM-PD on CT in order to determine patterns that differ from those in patients with bronchiectasis without NTM-PD. This might help practitioners to identify patients who require regular testing or therapy for NTM. Furthermore, radiologic changes after successful therapy were evaluated to serve as imaging biomarkers for the evaluation of the longitudinal course in the future.

## 2. Materials and Methods

### 2.1. Study Population

This is a retrospective, single-centre study, which was approved by our Internal Review Board (No. 8953_BO_K_2020). We included all patients between the ages of 18 and 80 years who visited our specialized Outpatient Department between 2011 and 2020 on the basis of their bronchiectasis, and who underwent a CT examination in our Radiology Department during this time. Most patients of our Outpatient Department had received external CT scans. Patients with NTM-PD, according to the ATS/IDSA diagnostic criteria [14], were also included if they had undergone a CT examination of sufficient quality at another medical institution. These criteria demand confirmatory microbiologic evidence of NTM infection in the context of clinical and radiographic findings suggestive of NTM pulmonary disease. All patients had received extensive work-up in accordance with the ERS guidelines in order to determine the aetiology of their bronchiectasis [13]. Data on clinical factors, including age, sex, body mass index (BMI), medical history, and duration of NTM-PD treatment, were collected from the electronic medical record system. All sputum-producing patients are examined for mycobacteria at every visit, in accordance with our outpatient routine. In case no spontaneous sputum could be sampled, hypertonic saline inhalation was provided.

### 2.2. CT Data Acquisition

The CT examinations were performed during clinical stability and no CT during acute exacerbation was evaluated. Most CT examinations were performed in our Radiology Department. CT examinations were obtained with a 64 row MDCT (Lightspeed VCT, GE Healthcare, Milwaukee, WI, USA) or a dual source CT (*n* = 78; Somatom Force, Siemens, Erlangen, Germany). All CT data were acquired volumetrically, using a standard dose protocol with 120 kV. CT data were reconstructed with a slice collimation of 1.25 mm and an interval of 1 mm. If required, an intravenous contrast medium was used for the particular clinical situation and 37 CT examinations were performed externally in patients with NTM-PD, using differing protocols and a slice thickness varying from 1.25 mm to 5 mm. CT with insufficient quality due to a slice thickness of >5 mm or too severe motion artefacts were excluded.

### 2.3. CT Features and Semiquantitative Scoring 

An evaluation of the CT examinations was conducted by a radiologist with 12 years of clinical experience in reading and evaluating chest CT (SD), and who was also blinded to the diagnosis. Bronchiectasis was diagnosed according to the criteria described by Naidich [18]. The Reiff-score [19] was used for the evaluation of bronchiectasis. Therefore, each lobe (with the lingula considered as a separate lobe) was scored for the extent of involvement (0 = none, 1 = one or partial segment, 2 = two or more segments); severity of bronchial dilatation (0 = normal, 1 = less than twice the diameter, 2 = 2–3 times the diameter, and 3 = more than 3× the diameter of the adjacent pulmonary artery); severity of the bronchial wall thickening (0 = normal, 1 = half the diameter, 2 = 0.5–1× diameter, and 3 = more than 1× the diameter of the adjacent pulmonary artery); type of bronchiectasis (1 = cylindrical, 2 = varicose, or 3 = cystic according to Reid [20]). Later, the lobar distribution of bronchiectasis (0 = widespread, 1 = predominantly upper lobe, 2 = predominantly middle lobe, 3 = predominantly lower lobe, 4 = middle and lower lobes equally involved, or 5 = unclassifiable) was registered [19]. In addition, a scoring for NTM-PD according to Kim et al. was performed [21]. Therefore, on an ordinal scale from 0–3, bronchiectasis including mucus plugging, cellular bronchiolitis, cavity, nodules, and consolidations were registered. Regarding bronchiectasis and cellular bronchiolitis, the severity (0 = normal, 1 = less than twice the diameter, 2 = 2–3 times the diameter, and 3 = more than 3× the diameter of the adjacent pulmonary artery) was registered. Bronchiectasis, mucus plugging and cellular bronchiolitis (0 = none, 1 = 1–5 segments, 2 = 6–9 segments, 3 = more than 9 pulmonary segments) were registered with regard to their extent. Cavities were scored for the diameter (1 = <3 cm, 2 = 3–5 cm, 3 = >5 cm), wall thickness (1 = <1 mm, 2 = 1–5 mm, 3 = >5 mm) and extent (1 = 1–3 cavities, 2 = 4–5 cavities, 3 = >5 cavities). Nodules (1 = 1–5 segments, 2 = 6–9 segments, 3 = >9 segments) and consolidations (1 = <3 segments, 2 = 3–5 segments, 3 = >5 segments) were scored according to the number of involved segments [21].

Furthermore, collateral findings were registered. Therefore, mucus plugging, tree-in-bud, peripheral and central consolidations, peripheral and central ground-glass opacities, interlobular septal thickening and intralobular lines were scored (0 = none, 1 = 1–3 bronchopulmonary segments involved, 2 = >3 bronchopulmonary segments involved) for the entire lung. Mosaic attenuation, atelectasis and emphysema were classified as present/absent. Subsequently, it was specified if bronchiectasis was predominant in the middle and lower lobes and if both mucus plugging and tree-in-bud were present in more than three segments. All terms were used according to the definition of the Fleischner Society [22]. At last, it was registered if there was a (sub)total atelectasis or a previous resection of a lower or middle lobe/lingula. 

In patients with an available consecutive CT scan after microbiological cure of NTM-PD, imaging findings were also scored according to Kim at al. as described before [21]. Microbiological cure was defined based on the absence of a positive culture result for at least 12 months of continuous treatment, following sputum conversion.

### 2.4. Statistical Analysis

The IBM SPSS Statistics (version 26.0, IBM Corp., Armonk, NY, USA) statistical software program was used to analyse the data. Binary variables are shown as numbers (*n*) and percentages (%). Category variables are shown as mean and median. Imaging features were compared between patients with and without NTM-PD using the Mann–Whitney U-test for categorical and the Chi-square test for binary variables. For comparison of the two consecutive CT scans, the Wilcoxon test was performed.

## 3. Results

### 3.1. Baseline Characteristics

In our study, 128 patients with bronchiectasis were included, 36 of them with NTM-PD. Patient characteristics are shown in Table 1. 

In 12 patients, follow-up CT scans with microbiological cure after treatment completion were available for analysis. In 14 patients no NTM therapy was received (“watchful waiting”) and 7 had undergone unsuccessful therapy. The remaining 3 patients had not received any evaluable follow-up CT.

### 3.2. CT Features of NTM-PD and Non-NTM Bronchiectasis

Results regarding the involvement, bronchial dilatation, and bronchial wall thickening, according to Reiff [19], are given in Table 2. 

In patients with NTM-PD, there was significantly less bronchial wall thickening (mean 0.42 in the left lower lobe/0.56 in the right lower lobe in patients with vs. 1.11/1.14 in patients without NTM-PD, both *p* < 0.001) and bronchial dilatation (mean 0.89 in the loft lower lobe 1.00 in the right lower lobe in patients with vs. 0.57/1.53 in patients without NTM, *p* = 0.001/0.007) in the lower lobes, compared to patients with bronchiectasis without NTM-PD. The lobar distribution differed significantly between patients with and those without NTM-PD: bronchiectasis in patients with NTM-PD was found predominantly in the middle lobe (56% of patients with versus 13% of patients without NTM-PD, *p* < 0.001), whereas non-NTM bronchiectasis was found predominantly in the lower lobes (25% of patients with versus 55 % of patients without NTM-PD, *p* = 0.002) (Table 3). Furthermore, bronchiectasis presented itself rather asymmetric (22% of patients with vs. 13% of patients without NTM-PD, *p* = 0.013) and was located peripherally (72% of patients with vs. 11% of patients without NTM-PD, *p* < 0.001) in patients with NTM-PD (Table 3).

Regarding cavities, the diameter (mean 0.28 vs. 0.08 in patients with and without NTM-PD, respectively, *p* = 0.038), wall thickness (mean 0.42 vs. 0.10 in patients with and without NTM-PD, *p* = 0.019) and extent (mean 0.25 in patients with NTM-PD vs. 0.09 in patients without NTM, *p* = 0.016) were significantly more accentuated in patients with NTM-PD. Other prominent collateral findings in patients with NTM-PD included the extent of bronchiolitis (mean 1.08 vs. 0.76 in patients with and without NTM-PD, *p* = 0.032), as well as small (mean 1.47 vs. 0.17 in patients with and without NTM-PD, *p* < 0.001) and large (mean 0.53 vs. 0.03 in patients with and without NTM-PD, *p* < 0.001) nodules. Moreover, patients with NTM-PD showed significantly less peripheral ground-glass opacities (mean 0.42 vs. 0.80, *p* = 0.003), interlobular thickening (mean 0.28 vs. 0.68, *p* = 0.001), and intralobular lines (mean 0.11 vs. 0.67, *p* < 0.001) compared to patients without NTM-PD (Table 4).

**Figure 1:**
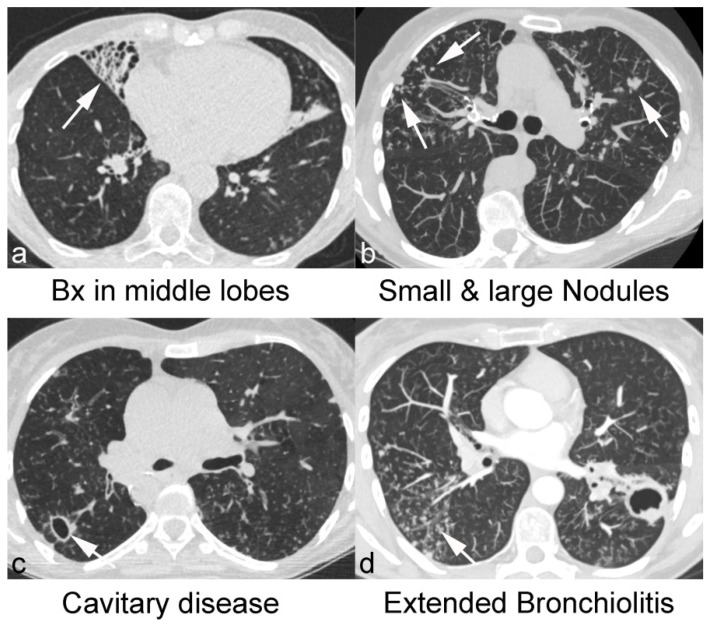
CT findings in patients with NTM-PD.

Figure 1 shows the characteristic CT findings in patients with NTM-PD.

### 3.3. Difference of CT Findings before and after Microbiological Cure Completion

Follow-up CTs were available for reassessment in 12 patients of the study cohort who had successfully completed NTM-PD treatment. These patients’ radiologic treatment response was evaluated according to the differences between the initial and the end-of-treatment CT score, in accordance with the extended published score by Kim et al. [21]. The sum score decreased from mean 10.92 to mean 8.67, however this change was not statistically significant (*p* = 0.073) (Table 5).

**Figure 2:**
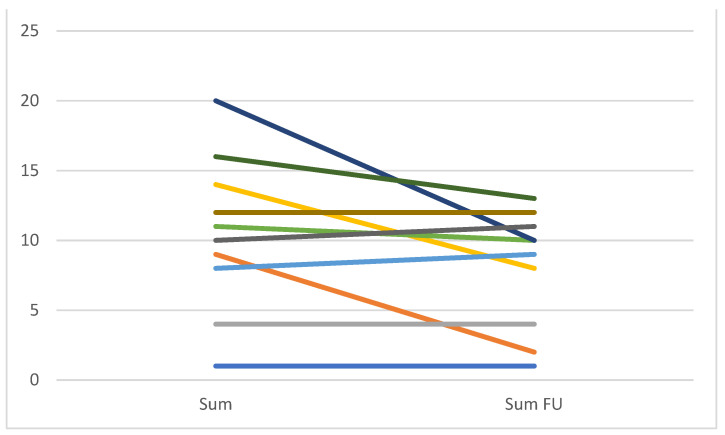
The NTM-CT score changes before and after successful NTM-PD treatment.

The only significant change in the analysis was a smaller scope of bronchiolitis (mean 1.17 vs. 0.50 in patients with and without NTM-PD, respectively, *p* = 0.038) after microbiological cure. Considering patients individually, the score decreased ≥3 points in 5 cases (Figure 2).

## 4. Discussion

Our results suggest that the CT patterns in patients with NTM-PD differ significantly from those in patients with bronchiectasis without NTM-PD. The predominance of bronchiectasis presented in the middle lobes, in addition to more small and large nodules indicate NTM bronchiectasis. Cavities turned out to be larger, with an extended wall thickness. Whereas patients without NTM-PD showed peripheral ground-glass opacities and interstitial changes more often. In addition, we observed a trend towards a lower CT score in the follow-up CT after successful NTM-PD treatment, and especially, the considerable resolution of bronchiolitis.

Various aetiologies exist which may result in bronchiectasis, including post-infective, chronic obstructive pulmonary disease, connective tissue disease, inherited disorders and infections including NTM-PD [23]. CT examination can provide valuable information on the aetiology of bronchiectasis [24]. In outdated studies, a correct aetiological diagnosis with CT could be made in less than 2/3 of patients [19,25,26]. Nevertheless, technical advances have been made in CT imaging during the last decade. A minimum bundle of aetiological tests in adults with a recent diagnosis of bronchiectasis is suggested in the European Respiratory Society (ERS) guidelines for management of adult bronchiectasis, along with identification of defined aetiologies, resulting in targeted therapeutic interventions such as immunoglobulin replacement, corticosteroids, and/or antifungal or antibiotic treatment [13]. The NTM-Network European Trials group (NTM-NET) recently called for greater awareness regarding the need for NTM screening in patients with bronchiectasis [12], especially regarding the improvement of NTM screening prior to the initiation of macrolide maintenance therapy. Despite its benefits, this long-term therapy may increase the risk of macrolide resistant NTM without appropriate sputum before initiating the therapy. The potential of CT as a predictive tool for identifying a patient’s underlying condition is currently underused in the field of bronchiectasis. To address this problem, a recently published Primary Ciliary Dyskinesia (PCD)-CT score in adults with bronchiectasis provided the first validated CT score with good sensitivity and specificity (83% respectively 83%). The score should guide physicians in identifying adult bronchiectasis patients who require further diagnostic workups for PCD [24]. The aim of the present study was to underline the usefulness of CT scans to identify concomitant NTM-PD in bronchiectasis. The answer to the question of whether NTM-PD is the aetiology, or an infectious complication of bronchiectasis is complex. It can only be answered on an individual basis and needs to consider the patients’ history, as well as sequential mycobacterial cultures and CT scans which are often not available.

The review by Anjos and co-workers mentions radiological findings derived from 18 articles on NTM-PD, including pulmonary cavitation (88.9%), bronchiectasis (77.8%), and pulmonary nodules (55.6%) [27]. The middle lobe and lingual were involved predominantly [27]. Eisenberg et al. evaluated differences between 32 patients with bronchiectasis and NTM-PD and 210 patients with bronchiectasis without NTM-PD [28]. In this study, people with NTM-PD showed a higher number of pulmonary segments involved with bronchiectasis, a higher extent of mucoid impactions, more tree-in-bud opacities, and more collapse/consolidation. The modified Bhalla score resulted in a lower score among people with NTM-PD, compared to non-NTM bronchiectasis. The combination of radiological features with advanced age and female gender predicted NTM-PD in patients with bronchiectasis. This prediction tool could not be applied efficiently in patients with Pseudomonas aeruginosa coinfection. Concerning the tree-in-bud opacity, the results are in complete concordance with ours. Additionally, collapse was more frequent in patients with NTM-PD in both studies; however, without any statistical significance in our present study. Similarly, Eisenberg et al. could not find a statistically significant different distribution in patients with and without NTM-PD. Nevertheless, the percentage of patients with a predominance of bronchiectasis in the middle lobes was higher in patients with NTM-PD, while the lower lobes were predominantly affected in patients without NTM-PD in our study cohort.

CT in NTM-PD can be used in a wide variety of clinical settings, e.g., for supporting the diagnosis of NTM-PD, disease monitoring (“watchful waiting”) and to determine the timing of treatment initiation [28,29,30,31]. However, little is known about the dynamics of CT findings during NTM treatment. The optimal duration of therapy for NTM-PD is not currently known. The guidelines suggest a treatment duration of at least 12 months after culture conversion for NTM-PD due to Mycobacterium avium complex with very low certainty in estimates of effect [14,32]. Radiological changes during treatment are not implemented in the successful treatment definitions [14,32,33]. A previous observational retrospective study in 72 patients with nodular/bronchiectatic MAC lung disease reported the following risk factors for disease progression: low body mass index, pulmonary cavities, consolidations, and macrolide resistance at presentation [34]. Our study showed that after microbiological cure, bronchiolitis was significantly reduced in these patients; however, the overall radiological findings improved only slightly. We observed considerable interindividual variation and no radiographic changes despite microbiological cure in some patients. These different courses could indicate factors associated with future recurrence or relapse of NTM-PD. Choi and colleagues identified CT findings that predict recurrence after successful treatment of NTM-PD [35]. Patients with recurrent NTM-PD showed higher CT scores for bronchiectasis, nodules, and consolidation. The authors concluded that patients with higher scores should be monitored closely for the recurrence of NTM-PD [35]. Structural lung abnormalities are predisposing host factors for NTM infection and recurrence [36] which may be one of the reasons why patients with bronchiectasis have an increased risk of developing NTM-PD. Respectively, quantitative chest CT findings serve as a diagnostic tool for the identification or confirmation of NTM-PD, as well as an imaging biomarker for evaluation of the longitudinal course.

The major limitation of our study is the small number of patients, especially among those after microbiological cure completion. Here, further studies with more patients are required. Another limitation of our study is the use of different CT scanners and different CT protocols. However, we screened the CT studies for image quality and excluded studies that did not adhere to the quality criteria set forth by the American Association of Physicists in Medicine [37].

In conclusion, the CT imaging features of adult patients with bronchiectasis differed between patients with and without NTM-PD. Our findings may help clinicians to identify or confirm patients with NTM-PD according to ATS/IDSA (2007) and ATS/ERS/ESCMID/IDSA (2020) clinical practice guidelines [14,32]. Further research is needed regarding the use of CT as a potential imaging biomarker for the evaluation of treatment response.

## Figures and Tables

**Table 1:** Characteristics for patients with and without NTM-PD.

Characteristics		Non-NTM (*n* = 92)	NTM (*n* = 36)
Sex-n (%)	Male	40 (43)	10 (28)
	Female	52 (57)	26 (72)
Age at CT–Median (IQR)		50 (33–64)	65 (61–75)
Aetiology-n (%)	Idiopathic	26 (28.3)	8 (22.2)
	PCD/Kartagener	11 (12)	0
	Asthma/ABPA	10 (10.8)	0
	Immunodeficiency	8 (8.7)	1 (2.8)
	COPD/A1AT	8 (8.7)	0
	Postinfectious	4 (4.3)	3 (8.3)
	CFTR-related disorder	4 (4.3)	1 (2.8)
	GvHD	3 (3.3)	0
	Others	7 (7.6)	0
	NTM-PD		23 (63.9)
Sputum microbiology-(%)	MAC		32 (88.9)
	*M. abscessus* subsp*. abscessus*		2 (5.6)
	*M. kansasii*		2 (5.6)
	*Pseudomonas aeruginosa*	26 (28.2)	6 (16.7)
	*Aspergillus* sp.	17 (18.5)	4 (11.1)

MAC = *M.avium* 23 (63.9), *M. intracellulare* 6 (16.7), *M. chimaera* 3 (8.3). Abbreviations: *n* = number, SD = standard deviation, IQR = interquartile range, CT = computed tomography, BSI = bronchiectasis severity index, ABPA = allergic bronchopulmonary aspergillosis, COPD = chronic obstructive pulmonary disease, A1AT = alpha-1 antitrypsin deficiency, GvHD = graft versus host disease, NTM = nontuberculous mycobacteriosis, CFTR = cystic fibrosis transmembrane conductance regulator, FEV1 = forced expiratory pressure in 1 s, FVC = forced vital capacity, NTM-PD = nontuberculous mycobacterial pulmonary disease.

**Table 2:** Results for the evaluation, according to the Reiff-Score.

Feature		OVERALL			NTM			Non NTM			Group Comparison *
		*n*	Mean	Median	*n*	Mean	Median	*n*	Mean	Median	*p*-Value
Involvement	right upper lobe	128	1.07	1.00	36	1.25	1.00	92	1.00	1.00	0.172
	middle lobe	120	1.44	2.00	35	1.54	2.00	85	1.40	1.00	0.336
	right lower lobe	126	1.44	2.00	36	1.25	1.50	90	1.51	2.00	0.108
	left upper lobe	127	0.85	1.00	36	0.97	1.00	91	0.80	0.00	0.347
	lingula	126	1.17	1.00	36	1.36	2.00	90	1.10	1.00	0.119
	left lower lobe	122	1.36	2.00	36	1.17	1.50	86	1.44	2.00	0.142
Bronchial dilatation	right upper lobe	128	1.09	1.00	36	1.19	1.00	92	1.05	1.00	0.298
	middle lobe	119	1.54	1.00	35	1.66	2.00	84	1.49	1.00	0.395
	right lower lobe	126	1.38	1.00	36	1.00	1.00	90	1.53	1.00	0.007
	left upper lobe	127	0.76	0.00	36	0.78	1.00	91	0.75	0.00	0.507
	lingula	125	1.27	1.00	36	1.33	1.00	89	1.25	1.00	0.662
	left lower lobe	122	1.37	1.00	36	0.89	1.00	86	1.57	2.00	0.001
Bronchial wall thickening	right upper lobe	127	0.54	0.00	36	0.56	0.00	91	0.54	0.00	0.810
	middle lobe	115	0.80	1.00	32	0.81	1.00	83	0.80	1.00	0.802
	right lower lobe	123	0.97	1.00	36	0.56	0.00	87	1.14	1.00	<0.001
	left upper lobe	127	0.33	0.00	36	0.42	0.00	91	0.30	0.00	0.369
	lingula	122	0.66	1.00	33	0.67	1.00	89	0.65	1.00	0.924
	left lower lobe	121	0.90	1.00	36	0.42	0.00	85	1.11	1.00	<0.001

Results of the Reiff-Score for all patients with bronchiectasis and separately for those with and without NTM-PD. * Group comparisons between patients with and without NTM-PD were performed using the Mann–Whitney U-test. Not all features could be evaluated in all lobes due to lobe resections and atelectasis.

**Table 3:** Results of the distribution of bronchiectasis.

		Overall (N = 128)	NTM (N = 36)	Non-NTM (N = 92)	*p*-Value *
Lobar distribution	widespread	25 (20)	4 (11)	21 (23)	0.214
	predominantly upper	10 (8)	3 (8)	7 (8)	1.000
	predominantly middle	32 (25)	20 (56)	12 (13)	<0.001
	predominantly lower	60 (47)	9 (25)	51 (55)	0.002
	NE	1 (1)	0 (0)	1 (1)	
Symmetry	symmetric	108 (84)	28 (78)	80 (87)	0.008
	asymmetric	20 (16)	8 (22)	12 (13)	0.013
Site	central	69 (54)	1 (3)	68 (74)	<0.001
	peripheral	36 (28)	26 (72)	10 (11)	<0.001
	mixed	21 (16)	7 (19)	14 (15)	0.599
	NE	2 (2)	2 (6)	0 (0)	

Results of the distribution of bronchiectasis, according to Reiff, for all patients with bronchiectasis and separately for those with and without NTM. Numbers are shown as n (percentage). * Group comparisons between patients with and without NTM were performed using Fischer’s exact test. NE = could not be evaluated.

**Table 4:** Results of the collateral findings in CT.

		Overall (N = 128)		NTM-PD (N = 36)		Non-NTM (N = 92)		Group Comparison *p*-Value *
		Mean	Median	Mean	Median	Mean	Median	
Cavity	diameter	0.13	0.00	0.28	0.00	0.08	0.00	0.038
	wall thickness	0.19	0.00	0.42	0.00	0.10	0.00	0.019
	extent	0.13	0.00	0.25	0.00	0.09	0.00	0.016
Mucus plugging extent		1.16	1.00	1.03	1.00	1.22	1.00	0.117
Bronchiolitis	severity	1.33	2.00	1.58	2.00	1.23	2.00	0.084
	extent	0.85	1.00	1.08	1.00	0.76	1.00	0.032
Nodules	small	0.54	0.00	1.47	2.00	0.17	0.00	<0.001
	large	0.17	0.00	0.53	0.50	0.03	0.00	<0.001
Atelectasis extent		0.78	1.00	0.89	1.00	0.74	1.00	0.241
Consolidations peripheral extent		0.53	0.00	0.53	0.00	0.53	0.00	0.916
Consolidations central extent		0.05	0.00	0.03	0.00	0.05	0.00	0.524
Ground-glass peripheral extent		0.70	1.00	0.42	0.00	0.80	1.00	0.003
Ground-glass central extent		0.17	0.00	0.08	0.00	0.21	0.00	0.116
Interlobular thickening extent		0.57	0.00	0.28	0.00	0.68	1.00	0.001
Intralobular lines extent		0.52	0.00	0.11	0.00	0.67	1.00	<0.001

Results of the collateral findings in CT with an ordinary scale for all patients with bronchiectasis and separately for those with and without NTM-PD. Mean values are given for the total patient group and separately for patients with and without NTM-PD. * Group comparisons were performed using the Mann–Whitney U-test.

**Table 5:** Follow-up data in CT after microbiological cure.

		Baseline Mean (Median)	Follow-Up Mean (Median)	*p*-Value *
Sum		10.92 (10.50)	8.67 (10.00)	0.073
Bronchiectasis	sum	4.92 (5.00)	4.92 (5.50)	1.000
	severity	2.25 (3.00)	2.25 (3.00)	1.000
	extent	1.75 (1.50)	1.83 (2.00)	0.317
	Mucus plugging	0.92 (1.00)	0.83 (1.00)	0.317
Cavity	sum	1.33 (0.00)	0.75 (0.00)	0.102
	diameter	0.42 (0.00)	0.25 (0.00)	0.317
	wall thickness	0.67 (0.00)	0.33 (0.00)	0.102
	extent	0.25 (0.00)	0.17 (0.00)	0.317
Bronchiolitis	sum	2.75 (2.50)	1.33 (0.00)	0.045
	severity	1.58 (1.50)	0.83 (0.00)	0.071
	extent	1.17 (1.00)	0.50 (0.00)	0.038
Nodules		1.42 (1.50)	1.33 (1.50)	0.564
Consolidation		0.50 (0.50)	0.33 (0.00)	0.157

Differences of CT findings in 12 patients with NTM before and after microbiological cure. * Group comparisons between patients with and without NTM-PD were performed using the Chi-square test for binary variables.
